# Oral innate immunity in patients with type 2 diabetes mellitus in a tertiary hospital in Ibadan Nigeria: a cross-sectional study

**DOI:** 10.11604/pamj.2022.43.134.34287

**Published:** 2022-11-14

**Authors:** Olatunde Ayodeji Olayanju, Izuchukwu Nnachi Mba, Nnaemeka Elvis Awah, Olukayode Olubumi Akinmola, Ekitumi Ofagbor, Onyiye Okonkwo, Olanrewaju Ezekiel Olasehinde, Akinbayo Abdul-Razaq Akintunde, Fayeofori Abbiyesuku

**Affiliations:** 1 Department of Chemical Pathology, Ben Carson (Snr) College of Health and Medical Sciences, Babcock University, Ilisan, Nigeria,; 2 Department of Chemical Pathology, Nile University of Nigeria, Abuja, Nigeria,; 3 Department of Chemical Pathology, University College Hospital, Ibadan, Nigeria,; 4 Department of Chemical Pathology, Lagos University Teaching Hospital, Lagos, Nigeria,; 5 Royal Free London, National Health Service (NHS) Foundation Trust, London, England,; 6 Department of Anatomical Pathology, University College Hospital, Ibadan, Nigeria

**Keywords:** Lysozyme, histatins, diabetes, innate immunity, saliva

## Abstract

**Introduction:**

diabetes mellitus is associated with a high prevalence of oral infections. However, it is unclear how diabetes impacts oral innate antimicrobial proteins. This study evaluated salivary lysozyme and histatins, two major innate antimicrobial proteins, in patients with diabetes and non-diabetic controls.

**Methods:**

a cross-sectional study where salivary lysozyme and histatins were measured alongside plasma glucose levels. Values of the salivary proteins were compared between the two groups; their association with glucose levels was also established using correlation and regression analysis.

**Results:**

one hundred and fifty-one participants were recruited for this study, 85 (56.3%) of them had type 2 diabetes mellitus with a median fasting plasma glucose of 108.8 mg/dl (IQR 91.2-134.8) while the remaining 66 (43.7%) healthy non-diabetic controls had a median random plasma glucose of 101 mg/dl (IQR 89-112). The median salivary lysozyme was 32.5 ng/ml (IQR 25.0-39.6) in the group with diabetes and 36.4 ng/ml (IQR 31.4-42.1; p=0.01) in the non-diabetic control group. The median salivary histatins was 9.2 ng/ml (IQR 7.6 -10.2) in the group with diabetes and 14.7 ng/ml (IQR12.8-16.5; p<0.001) in the non-diabetic control group. Salivary lysozyme (r = -0.127; p =0.163) and histatins (r = -0.025; p = 0.424) were both negatively correlated with plasma glucose levels, and logistic regression showed that patients with diabetes are more likely to have lower levels of salivary lysozyme (0.957; p=0.013) and histatins (0.527; p<0.001).

**Conclusion:**

patients with diabetes had reduced levels of salivary lysozyme and histatins, this could provide an insight into the associated high oral infection rates.

## Introduction

Diabetes mellitus is a leading non-communicable disease cause of death in the world and the prevalence has continued to increase globally [1,2]. Though treatment options are relatively affordable, non-compliance to treatment is high among affected patients [3-5]. Consequently, most patients develop complications which worsen treatment success and quality of life [6]. Retinopathy, neuropathy and nephropathy are widely documented complications of diabetes [7], while oral complications are mostly overlooked in routine clinical evaluation, though they have critical implications for affected individuals [8,9].

A wide spectrum of oral disorders has been associated with diabetes [10-12]. This association is attributed to the characteristic dysfunction of both the adaptive and the innate immune system. Defective phagocytosis by macrophages and dysfunctional antibody production contribute to weak immunological responses [13,14]. Furthermore, the flow rate of saliva, which serves as the transport medium within the oral cavity, is substantially reduced in the patients with diabetes thereby compounding the ineffective immune response [15].

The oral cavity is estimated to be harbouring over 400 different species of bacteria but their pathogenicity is curtailed by the immunological cells and antimicrobial proteins within the saliva which baths the cavity [16]. More than 300 proteins have been isolated from the saliva; only a few of those proteins have antimicrobial activities [17-19]. Being the first line of protection against invading microbes, the innate antimicrobial proteins in the saliva are critical in maintaining the integrity of the oral cavity. A few of them like lysozyme, histatins, and lactoferrin have all been described to play significant immuno-protective roles in the oral cavity [20]. This protective functionality is believed to be compromised in the patients with diabetes [21].

Lysozyme is generally believed to prevent the overgrowth of potentially dangerous organism while allowing the growth of harmless and beneficial species in the oral cavity [22]. Histatins on the other hand are widely known to have a broad-spectrum antifungal activity in the oral cavity [23,24]. These two innate antimicrobial proteins, amongst the others are expected to provide the required balance between normal flora and pathogenic organisms in the oral cavity. It is however not clear if diabetes mellitus causes a reduction in their levels. Thus, the aim of this study is to compare the levels of salivary lysozyme and histatins which are major innate antimicrobial proteins between patients with type 2 diabetes mellitus and healthy non-diabetic controls.

## Methods

**Study design and setting:** this was a cross-sectional study on patients with type 2 diabetes mellitus and healthy non-diabetic controls. The study was conducted at the Metabolic Research Ward of the Chemical Pathology Department at the University College Hospital, a tertiary hospital located in Ibadan, Nigeria. The study was conducted between June and September 2020.

**Study population:** participants in this study were patients with type 2 diabetes attending the outpatient clinic and healthy non-diabetic controls who were consenting members of staff of the same hospital. Only participants above the age of 18 were recruited and people on steroids, immunosuppressive drugs and cigarette smokers were exempted from the study. Sample size was determined using the formular for cross-sectional study with quantitative variables [25].

**Data collection:** demography and clinical data were obtained from the participants using a self-administered questionnaire following their informed consent.

**Sample processing and laboratory analysis:** all participants were asked to rinse their mouth with clean water and wait for about ten minutes before providing unstimulated saliva samples between 8 a.m. and 11 a.m. on the days of their recruitment. The samples were collected passively into clean universal bottles and transferred to the laboratory from where they were decanted into clean serum bottles. Sample processing included centrifugation at 3000 radians/minute for 15 minutes, transfer of the clear supernatant into clean 1.5 ml Eppendorf tubes for storage at -20°C until the time of laboratory analysis. Salivary lysozyme and histatins were measured using enzyme-linked immunosorbent assay technique according to the manufacturer´s instruction (Melsins Medicals, Changchun, China). About 2 mls of fasting venous blood sample was also collected from all patients with diabetes, while the healthy non-diabetic controls provided the same volume of random venous blood sample for glucose estimation using Landwind C100 autoanalyser according to manufacturer´s instruction. The process was completed within three months.

**Statistical analysis:** the diabetes effect on the salivary antimicrobial proteins was determine by comparing values between the group of patients with diabetes mellitus and the healthy non-diabetic control group. Normally distributed data were reported as mean ± standard deviation, while data that were not normally distributed were reported as median (interquartile range); categorical data were reported as frequency (percentage). Comparison between the two groups was done using independent sample T-test and Mann-Whitney U test as appropriate, depending on nature of data. Relationship between selected variables and diabetic status was determined using logistic regression analysis, and variables with significant p-values were used to compute a multivariate regression model. P values less than 0.05 were considered significant, and analysis was done using Statistical Package for Social Sciences (SPSS) analytical software version 26 (IBM Corporation, Armonk, NY, USA).

**Ethical consideration:** ethical approval was obtained from the University of Ibadan/University College Hospital Research Ethics Committee. The right of confidentiality of all participants was protected all through the study. The benefits of potential study findings were explained to all participants who voluntarily took part in the study, and no form of harm was inflicted on the subjects during this study.

## Results

**General characteristics of the study population:** a total of 151 participants met the criteria for inclusion in this study, their mean age was 56.9 ± 11.2 years and 76 (50.3%) of them were males. The participants consist of 85 patients with (56.3%) type 2 diabetes mellitus with a median fasting plasma glucose of 108.8 mg/dl (IQR 91.2-134.8) and 66 (43.7%) healthy non-diabetic controls with a median random plasma glucose of 101 mg/dl (IQR 89-112). There was no significant difference between the ages of the participants with diabetes compared to the control group (56.9 ± 12.9 vs 56.7 ± 8.5; p=0.98). There are, however, more males in the control group (48/66; 72.7%) than in the group with diabetes (28/85; 32.9%; p<0.01). Other demographic data are presented in Table 1.

**Table 1:** comparison of biodata between patients with diabetes and healthy non-diabetic control

Variables	Patients with diabetes (n=85)	Non-diabetic control (n=66)
Age (years)	56.9 ± 12.9	56.7 ± 8.5
Gender (male)	28 (32.9%)	48 (72.7%)
Weight (kg)	75 (IQR 63.8 - 83)	72 (63 - 81)
Height (m)	1.6 (1.55 - 1.65)	1.68 (1.60 - 1.75)
*BMI	29 (26.0 - 32.7)	26.3 (22.7 - 29.1)
#Tertiary education	52 (61.2%)	41 (62.1%)

BMI: body mass index

**Figure 1:**
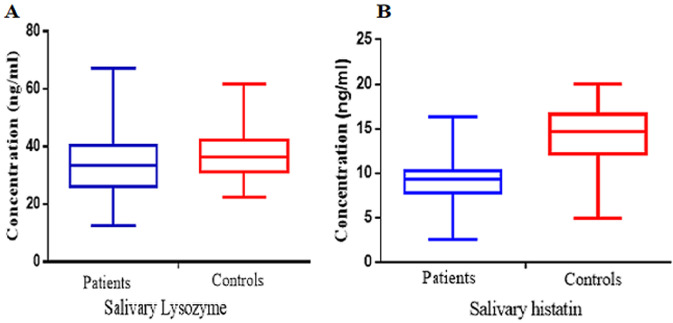
box and whisker plots of salivary lysozyme; A) and histatins; B) showing the comparison between the patients with diabetes and the healthy non-diabetic controls

**Antimicrobial proteins and tests of association:** the salivary lysozyme was significantly lower (p=0.01) in the patients with diabetes measuring a median 32.5 ng/ml (IQR 25.0-39.6) compared to 36.4 ng/ml (IQR 31.4-42.1) in the non-diabetic control group. The salivary histatins was also significantly lower (p<0.001) in the patients with diabetes measuring a median 9.2 ng/ml (IQR 7.6 -10.2) compared to 14.7 ng/ml (IQR12.8-16.5) in the non-diabetic control group (Figure 1).

Correlation analysis done showed that levels of salivary lysozyme (r = -0.127; p=0.163) and histatins (r = -0.025; p = 0.424) were both negatively correlated with plasma glucose levels in the patients with diabetes. The same pattern was found in the relationship between plasma glucose and salivary lysozyme and histatins in the healthy non-diabetic control (Table 2).

**Table 2:** correlation between plasma glucose and levels of salivary antimicrobial proteins

Variables	Lysozyme		Histatins	
	Correlation coefficient	p-value	Correlation coefficient	p-value
Healthy control	-0.215	0.043	-0.340	0.003
Patients with diabetes	-0.127	0.163	-0.025	0.424

Binary logistic regression analysis done to determine the relationship of selected variable with confirmed diabetic state essentially showed that patients with diabetes are more likely to have higher body mass index (BMI) (O.R 1.110 (1.028-1.198); p=0.008) and lower values of salivary lysozyme (OR 0.957 (0.924-0.991); p=0.013) and histatins (OR 0.527 (0.435-0.638); p<0.001). Multivariate analysis however, showed that only higher BMI (OR 1.153 (1.042-1.275); p = 0006) and lower histatins levels (OR 0.560 (0.455 - 0.689); P < 0.001) are directly associated with the diabetic status, as shown in Table 3.

**Table 3:** binary logistic regression analysis showing the relationship between selected variables and diabetes status

Variable	O.R (95% C.I)	p-values
**Univariate analysis**		
Age	1.000 (0.971 - 1.029)	0.98
BMI	1.110 (1.028 - 1.198)	0.008
Lysozyme	0.957 (0.924 - 0.991)	0.013
Histatins	0.527 (0.435 - 0.638)	<0.001
**Multivariate analysis**		
BMI	1.153 (1.042 - 1.275)	0.006
Lysozyme	1.032 (0.975 - 1.097)	0.28
Histatins	0.560 (0.455 - 0.689)	<0.001

BMI: body mass index

## Discussion

Diabetes mellitus is associated with impaired cellular and humoral immunological responses, and this is the basis for most local and systemic infections present in affected individuals. The oral mucosa of the patients with diabetes is particularly predisposed to several widely described infectious and inflammatory conditions [10]. This study sought to measure proteins in the saliva of patients with diabetes and establish a relationship. In this study, we found a reduced levels of lysozyme and histatins in the saliva of patients with diabetes compared to those of the healthy non-diabetic controls. We also found that lysozyme and histatins are negatively correlated with plasma glucose level, and that patients with diabetes are more likely to have reduced levels of these antimicrobial proteins.

The salivary lysozyme is one of the foremost antibacterial and anti-inflammatory agents in the oral cavity, its anti-inflammatory property has recently been employed in developing drugs to treat oral, skin and gastrointestinal diseases [26,27]. However, our study showed that salivary lysozyme level is significantly reduced in the patients with diabetes. This could provide an insight into the predominance of inflammatory and infectious disorders in the oral cavity of patients with diabetes. This finding corroborates what was reported in an earlier study done on adult patients with type 1 diabetes mellitus [28]. On the contrary, a similar study done in paediatric patients with diabetes reported a significantly higher salivary lysozyme levels compared to the control group, although the total salivary protein was significantly lower [29]. The age group of the children involved, and the presence of caries and other periodontal diseases may have constituted important confounders. It is however noteworthy that other studies showed that certain salivary proteins like immunoglobulins, defensins and cathelicidin are reportedly overexpressed in the oral cavity of patients with diabetes although they still do not protect them, and the mechanism behind this finding are not clear [19,30].

Salivary histatins are cationic peptides that protect the oral cavity against fungal agents, especially the candida species and has been described as a promising antifungal therapeutic agent in humans with oral candidiasis [31,32]. Levels of salivary histatins characteristically increases with periodontal disease progression, a normal response which is absent in patients with diabetes [33]. This was supported by the finding in this study which showed that patients with diabetes had significantly lower levels of histatins compared to the healthy non-diabetic controls. It is reasonably logical to attribute the increased prevalence of oral candidiasis in patients with diabetes to the reduced levels of the most potent antifungal agent in their saliva. A similar finding was reported, in a bid to establish the antifungal role of histatins in the oral cavity of HIV-infected individuals; although the exact mechanism was not clear, HIV infection also predisposes to an immunodeficiency state, just like diabetes [34].

It has been established that the level of glycaemic control usually affect the availability of salivary antimicrobial proteins [35]. In this study both lysozyme and histatins showed negative correlation with the plasma glucose level; this relationship was demonstrated in both groups. Furthermore, patients with diabetes showed higher propensity to have reduced level of these proteins when compared to the control group. This finding is in agreement with a previous study where 40 different salivary proteins were found to be significantly affected by patients´ glycaemic control [36]. This is particularly important in reiterating the role of glycaemic control in mitigating adverse oral complications that are generally associated with diabetes mellitus as evidence by scientific reports [37]. Ensuring that patients with diabetes comply with treatment regimen and regular clinic follow-up will help to improve glycaemic control and consequently reduced the prevalence of oral infection.

There are a few limitations to this study. A prospective study, rather than the cross-sectional type done in this study, would have allowed for the progressive monitoring of the changes in the salivary lysozyme and histatins over a long duration of treatment period, to substantiate the finding of the correlation and regression analysis in this study. Several other innate salivary anti-microbial proteins exist and may behave differently from lysozyme and histatins that we investigated in this study, however, both of them are key to the overall maintenance of the immunological balance in the oral cavity.

## Conclusion

This study showed that diabetes mellitus is associated with a significant reduction in the key innate antimicrobial proteins in the saliva of affected individuals. This may provide a reasonable explanation for the high propensity of oral infectious and inflammatory conditions in patients with diabetes. It is highly recommended that glycaemic control should be more diligently prioritised in order to salvage the protective antimicrobial proteins in the saliva of patients with diabetes, thereby reducing the prevalence of infection and inflammatory conditions.

### What is known about this topic

- There is a generalized impairment of immunological responses to microbial agents in individuals with diabetes mellitus;
- Diabetes mellitus is associated with higher prevalence of oral infections.

### What this study adds

- Patients with diabetes have lower levels of innate antimicrobial proteins lysozyme and histatins compared to non-diabetic people;
- The higher the plasma glucose levels, the lower the concentrations of these innate antimicrobial proteins in the saliva, thus predisposing to oral infections.
